# Consumers’ Decision-Making Process on Social Commerce Platforms: Online Trust, Perceived Risk, and Purchase Intentions

**DOI:** 10.3389/fpsyg.2020.00890

**Published:** 2020-05-15

**Authors:** George Lăzăroiu, Octav Neguriţă, Iulia Grecu, Gheorghe Grecu, Paula Cornelia Mitran

**Affiliations:** ^1^Faculty of Social and Human Sciences, Spiru Haret University, Bucharest, Romania; ^2^Department of Economic Sciences, Spiru Haret University, Bucharest, Romania

**Keywords:** consumer, decision-making process, social commerce platform, online trust, perceived risk, purchase intention

## Abstract

This study enhances the existing literature on the role of trust and online perceived risk in shaping consumer purchase decision-making in social commerce. The aim of this article is to investigate consumers’ purchase decision-making process, the determinant components of social commerce purchase intentions and attitudes, the effect of perceived risk on intention to go shopping in online settings, and consumer trust and buying behavior on online retailing platforms. The insights obtained from our research extend current knowledge as regards determinants of consumer attitudes and intentions toward online purchases, consumers’ perceived shopping risk and repurchase behavior when buying products online, and perceived consumer online trust and purchase decisions. Limited research has considered consumers’ decision-making processes on social commerce platforms by investigating how their perceptual attitudes, behavioral intentions, and immediate gratifications affect the purchase of products and services online. Our study addresses this gap and extends prior research by focusing on the relationship between online consumer purchase intention, social commerce adoption behavior, and consumers’ trust together with risk factors affecting online buying decisions, in light of the characteristics of source credibility. Our findings point toward important avenues of research on psychological determinants of consumer engagement in social media, decision mechanisms lying behind evaluation of prices, the types of perceived risk incurred, and online repurchasing behavior and intention on social commerce platforms. Subsequent directions should clarify whether adoption of mobile payment services may shape online consumers’ impulsive buying behavior and decision-making, especially under the influence of online product reviews.

## Introduction

Our study cumulates evidence that consumers’ decision-making process is a dynamic performance (Hollowell et al., 2019; Popescu and Ciurlău, 2019; Westbrook et al., 2019), but that the determinants of social commerce intention and online consumer choice behavior (Kovacova et al., 2019; Tuyls and Pera, 2019) can be clarified. We focus on how the antecedents of trust in social commerce can be predicted, developing on the role of social experience and information accuracy on consumer purchase decision.

Using exclusively empirical sources published recently (2017–2019) in Web of Science- and Scopus-indexed journals, our paper fills the gap in the literature by elucidating consumers’ evaluation, commitment, and choice throughout online decision-making, insisting on their cognitive processing behaviors and on how expectations shape their pre-purchase and post-purchase satisfaction.

## The Trust Building Mechanisms in Social Commerce

Guo et al. (2018) explain that the trust and perceived risk of retailing participants are pivotal grounds for the ascendancy of social commerce. Hall et al. (2017) hold that social media can be employed to integrate consistent views of consumers into the decision-making process. Abed (2018) insists that social influence and trust represent relevant components altering behavioral intention toward social commerce. Jeon et al. (2017) emphasize that utilitarian value and online trust thoroughly further the connection between perceived website interactivity and repurchase intention. Hansen et al. (2018) say that perceived risk and trust are decisive antecedents in end user decision-making, while risk-taking propensity influences behavioral intention directly. Moody et al. (2017) stress that trust and lack of confidence act on online relationships entailing electronic dealings. Ozturk et al. (2017) indicate that trust and perceived risk are related to loyalty. Fu et al. (2018) put it that both external and internal similarity considerably shapes consumers’ perceived usefulness, satisfaction, and trust transfer, which subsequently affect their shopping behaviors. Xu-Priour et al. (2017) report that trust and social interaction are favorably associated with online purchase intention. Pratono (2018) claims that trust in social commerce enables Internet retailers to achieve pricing and selling capabilities, which generates positive consequences on their performance. The adoption of social media in the management process does not influence the intensifying firm performance, unless the companies have confidence in social networks.

Pantano and Gandini (2017) argue that the retail environment is typified by a growing adoption of cutting-edge and interactive technologies contingent on advanced connectivity and pervasive and contactless systems that improve and reinforce consumer shopping practices. Digitally mediated in-store operation chiefly provides feedback on a demand for guidance and trust, and the types of sociality brought into play around it are intrinsically impermanent, low-intensity and publicity-led ways of joint action. Oliveira et al. (2017) remark that with the purpose of maximizing the entire trust of the end users in their business, Internet retailers should comprehend the way consumers perceive integrity, competence, and kindness. Perceived integrity happens when the end user comes to the conclusion that the Internet retailer is morally correct and behaves in good faith, without overcharging throughout sales transactions and sticks to his/her responsibilities, and is authentic. Perceived competence is attained when the end user thinks that the Internet retailer can manage sales transactions, being an expert in trade processes. Perceived kindness is when the end user regards as true that the Internet retailer proceeds in consumers’ best interest, and, if required, would endeavor to assist exemplarily. Kim and Peterson (2017) report that online trust is instrumental in consolidating confidence-related outcomes in social commerce. Ability, honesty, kindness, dependability, and predictability constitute important components of online trust. Molinillo et al. (2017) reveal that the affective and cognitive shopping practices favorably impact the level of contentment, the former also having a positive effect on trust. Consumer contentment with Internet retailers may be raised by both the hedonic and utilitarian values of social commerce. Mou et al. (2017) claim that perceived usefulness and trust are relevant at both the preliminary and subsequent phases in the end-user adoption of online services. Consumers’ concrete usage practices alter perceptions of convenience and shape the validation of their initial requirements.

Wongkitrungrueng and Assarut (2018) explain that symbolic value has a direct and indirect impact through trust in Internet retailers on consumer engagement. Utilitarian and hedonic values shape consumer engagement indirectly through end-user trust in products and in Internet retailers successively. Cui et al. (2018) hold that significant perceived security reinforces the assessment of website image and trust, end users having a relevant degree of creativity are likely to have confidence in websites, website image has a facilitating impact on perceived security and trust, while the latter is a link between website image and online loyalty. Yahia et al. (2018) stress that reputation and price advantage determine trust, but their implications are moderated by habits. Social dealings with the social commerce retailer and product differentiation diminish trust. Perceived ease of adoption of the platform, expediting conditions, hedonic reasons, and habits consolidate social commerce intent. Mou and Shin (2018) clarify that end users’ trust and perceived product quality and value affects social popularity. Time scarcity is decisive for perceived product quality and value, determining online consumers’ fixation attention. Lien et al. (2017) suggest that Internet retailers’ anxious attachment is adversely related to their trust in social commerce platforms. Relational embeddedness favorably impacts trust of Internet retailers in social commerce platforms.

Choi and Lee (2017) emphasize that user-generated content impacts consumers’ cognitive trust more than does marketer-generated content, and the implication of online product reviews in closed social network services on consumers’ cognitive and emotional trust is more significant than the implication in open social network services. The synergy between content generator types and social network service types influences consumers’ emotional trust. López-Miguens and Vázquez (2017) maintain that in social commerce, contentment, trust, and switching boundaries impact loyalty directly. Trust generates loyalty with contentment as a go-between. Website quality leads to loyalty facilitated by contentment and/or trust. As Cherrett et al. (2017) put it, online purchase behavior is facilitated in diverse regards by the lack of direct contact, displaying several important transaction issues associated with trust, responsibility for and cost of delivery, and swiftness. Fazal-e-Hasan et al. (2018) observe that objective attainment positively facilitates the link between expectation, end user contentment and trust, without influencing the impact of expectation on consumer affective commitment. Wu W. Y. et al. (2017) state that aesthetic appeal, personalization, functionality, and financial security shape consumer attitudes and trust in relation to a website. Increasing the top-of-mind awareness for online vendors’ brands may improve trust in their website. On Rubio et al. (2017)’s account, end users’ loyalty and trust constitute paramount characteristics for online vendors, as social commerce boosts competition and diminishes cost of change for consumers, making hard to retain the latter. Contentment with price levels, commitment to the store brands, and the perceived representation of the assortment constitute critical features of retail commitment and trust. Liu et al. (2018) note that end user-to-end user trust and end user -to-marketer trust favorably determine consumer engagement, which in turn has an impact on brand trust. The device usage facilitates the effect of consumer engagement on brand trust.

## Consumer Purchase Intention in Social Commerce

Mou et al. (2017) posit that end-user trust beliefs constitute a significant driver of e-service adoption. Chong et al. (2018) stress that consumers’ perceived effectiveness of institutional mechanisms detrimentally moderates online trust in retailers and repurchase intentions. Rahman et al. (2018) affirm that more than hedonic values, confidence, and privacy issues, utilitarian values favorably impacts buyers’ attitudes to online shopping. Yang et al. (2017) put it that mobility, trust in the service supplier, and security/privacy risk have an effect on the embracement of social commerce. Walsh et al. (2017) emphasize that an online vendor’s positive reputation may diminish end user’s risk and bring about trust, which subsequently fosters consumer commitment. Zhang et al. (2018) explain that consumers’ empowerment determines their perceived trust and contentment as regards their shopping practices, and their buying intention. Bahbouhi and Moussa (2017) insist that online exchanges frequently take place between unknown people who cannot count on previous behavior or the eventuality of subsequent interactions to lay the foundations of mutual trust. Hajli et al. (2017a) remark that consumers’ loyalty with regard to a brand improves as trust, commitment, and contentment level increase.

Xu-Priour et al. (2017) claim that polychronic orientation of consumers is likely to be positively associated with trust, social teamwork, browsing practice, and external locus of control, and incidentally to intention to purchase online via such consumer features. Oliveira et al. (2017) assert that the sources of consumer trust determine aspects such as expertise, integrity and kindness of the Internet retailer, that all determine the entire trust of an end user, inevitably affecting their online buying intention. Bashir et al. (2018) say that perceived financial risk of end users determines their online trust in web retailers and purchase intention. Consumers’ online purchase intention is affected by their degree of trust in Internet retailers. The consumers’ degree of trust ultimately determines their intent level in relation to searching and purchasing products from online stores, while speaking favorably of them to other end users. Mou and Cohen (2017) affirm that trust in the Internet retailer’s website acts on confidence in the online service supplier at both preliminary and subsequent phases. Interpretations of system and information quality are determined by trust, that, together with contentment, is relevant to continued usage intentions. Cao et al. (2018) notice that the trust transfer process favorably impacts the continuance intention of social commerce through contentment. Confidence in and perceived entitativity between online and mobile payments, together with perceived comparability, may positively shape trust in social commerce.

Zhang and Curley (2018) observe that explanation mode and availability, in addition to perceived personalization, impact consumers’ trust beliefs considerably in an online recommender agent and commitment to embrace its suggestions. Consequences of both the availability and mode of explanations on end users’ trust beliefs are facilitated by their perceived personalization of the online recommender agent that subsequently mediates the implications on use intention. Escobar-Rodríguez and Bonsón-Fernández (2017) put it that perceived value, trust, and creativity represent the essential components shaping online purchase intention, whereas time saving and perceived security constitute the chief antecedents determining perceived value and trust. Qin (2017) states that, as regards consumers’ intention to adopt social commerce, trust represents a construct of aspects of expertise, integrity, and kindness. Awareness and shared value constitute antecedents to trust aspects affecting them considerably. Culture significantly impacts consumers’ values, whereas trust is determined by cultural features. Liu and Guo (2017) find that universal access and awareness are unsuccessful in shaping trust directly but can impact purchase intention through social benefit. Reputation can have an effect on trust and social benefit immediately in social commerce markets. Bianchi et al. (2017) show that trust may generate positive attitudes in relation to social commerce as regards contentment, which subsequently can shape the intention to participate in online purchase behavior.

Vohra and Bhardwaj (2019) point out that trust to a certain extent enables the link between dynamic involvement and engagement. Shareef et al. (2018) posit that operational performance and trust may influence consumers’ purchase intention when shopping online. Sisson (2017) highlights that positivity and networking approaches have important relationships with features of trust, being useful in reestablishing integrity. On Bebber et al. (2017)’s reasoning, the constructs of data quality, lack of confidence, and perceived risk are antecedents of online purchase intention. As Robinson (2017) puts it, marketers may determine consumers’ decision making process on the perpetual shopping, by providing upsides, improving trust, and decreasing the perceived risks. Gibreel et al. (2018) write that familiarity and trust are a factor in facilitating exchange between vendors and end users and its favorable consequences in purchasers’ perceived convenience of social commerce platforms. Pappas (2018) reasons that trust in relation to online retailers, privacy, emotional states, and experience fuse to predict shoppers’ purchase intentions. On Pee et al. (2018)’s view, website usability functions as a lasting effect signal, determining the initial buying and repurchase intention. Malhotra et al. (2017) contend that perceived structural assurance operates as a trust-building mechanism for diminishing the adverse outcomes of psychological contract violation among online consumers.

## Effects of Perceived Risk on Purchase Intention in Social Commerce

On Wu J. et al. (2017)’s reading, trust is the essential component of consumer purchase intention when buying things on peer-to-peer temporary rental platforms where both hosts and renters do not know each other. Xie et al. (2017) state that trust has a beneficial impact on social norms, but that perceived risk adversely affects perceived behavior control. Li (2019) reveals that social presence, closeness, and informational reinforcement (but not emotional support or familiarity) exert intention over trust in product testimonials. On Sharma et al. (2019)’s account, online trust in companies seriously affects consumers’ trust and essentially their intention to participate in social commerce. Izogo and Jayawardhena (2018) show that moral correctness, dependability, and excellent reputation strengthen online shopping trust. Chen et al. (2017) point out that retailers can establish relational capital with purchasers by improving mutual trust, cooperation, and appreciation with them. Bleier et al. (2019) find that a manufactured item’s type and brand reliability influence the effect of each experience aspect on end users’ purchase decisions. Sullivan and Kim (2018) indicate that perceived value affects the experiences of online trust among purchasers and their commitment to buy again from the same website.

Sullivan and Kim (2018) demonstrate that trust, social commerce adoption, and product evaluation factors are essential in shaping repurchase intention: perceived quality is determined by the grasps of competitive price and website reputation, which subsequently have an effect on perceived value, that, together with website reputation and perceived risk, shape online trust, which acts upon repurchase intention. The impact of perceived convenience on repurchase intention is not relevant. Perceived value and online trust represent the main drivers of repurchase intention. Bashir et al. (2018) write that a mediating function of online trust operates in web retailers in the link between perceived financial risk and purchase intention. The perceived financial risk of online end users determines their trust in Internet retailers. Consumers’ intention to go shopping online is affected by their confidence in the web retailer. Trust in the Internet retailer represents confidence of a consumer in an Internet retailer’s ability, product-related knowledge, performance, marketing sufficiency, integrity, and payment procedures. On Choi et al. (2018)’s view, handling shopping risk constitutes a necessary condition to establishing business success in shopping destinations, because risk tends to shape perceived value and the selection of subsequent shopping destinations. Improving trust is important in preventing or decreasing perceived purchasing risk. Increased trust is likely to diminish shopping risk and eventually advance the view of a shopping destination as well-founded. On Martin (2018)’s reading, as information misuse represents a distinctly noticeable type of risk online, complying with privacy is frequently firmly associated with trust in end-user surveys. Breaches in privacy expectations cut down consumers’ trust in an Internet retailer, making difficult for the latter to rebuild confidence in its services and products. Han and Kim (2017) indicate that product and social/psychological risks are adversely related to end users’ trust and purchase intention, notwithstanding their level of product involvement, while financial risk is positively associated with trust and purchase intention.

Oliveira et al. (2017) show that trust is instrumental in influencing consumer purchase intentions: the entire trust that an end user has on an Internet retailer is determined by the reliability of the vendors, i.e., whether they are dependable and consumers have confidence in them, this relevantly impacting their online purchase intention. Consumers having notable overall trust display a considerable intention to go shopping online. Hajli et al. (2017b) posit that trust constitutes a critical concern in online shopping settings and in social commerce platforms because of the prominent function of peer-generated contents. Trust, awareness, social presence, and online shopping information seeking determine behavioral attitudes on social commerce platforms. Trust in an Internet retailer reinforces information seeking which subsequently boosts awareness of the platform and the perception of social presence, while awareness and social presence optimize buying intentions. Liu et al. (2017) contend that trust in social commerce websites consolidates purchase intention directly and strengthens the positive link between website appeal and purchase intention, simultaneously decreasing the positive link between product appeal and purchase intention. Akman and Mishra (2017) affirm that consumer intention is considerably and positively associated with perceived trust, social pressure, contentment, and cognizance in social commerce adoption. Hsu et al. (2018) point out due to the development of social media applications, online shopping is increasing noticeably in social commerce. Supplying outstanding quality website experience is essential to assist online consumers. The perceived system and service quality constitute relevant antecedents of consumer contentment that appreciably regulates trust, commitment, and purchase intention. Trust considerably influences commitment and both of them to a limited extent facilitate the link between contentment and purchase intention in social commerce setting.

## Conclusion

The empirical evidence we reviewed supports the belief that social platform users’ purchase intentions can be constituted taking into account the relationship between online trust and perceived risk. Limited research has considered consumers’ decision-making processes on social commerce platforms by investigating how their perceptual attitudes, behavioral intentions, and immediate gratifications affect the purchase of products and services online.

Our study addresses this gap and extends prior research by focusing on the relationship between online consumer purchase intention, social commerce adoption behavior, and consumers’ trust together with risk factors affecting online buying decisions, in light of the characteristics of source credibility. As limitations in the present article, our findings point toward important avenues of research on psychological determinants of consumer engagement in social media, decision mechanisms lying behind evaluation of prices, the types of perceived risk incurred, and online repurchasing behavior and intention on social commerce platforms. Subsequent directions should clarify whether adoption of mobile payment services may shape online consumers’ impulsive buying behavior and decision-making, especially under the influence of online product reviews.

## Author Contributions

All authors listed have made a substantial, direct and intellectual contribution to the work, and approved it for publication.

## Conflict of Interest

The authors declare that the research was conducted in the absence of any commercial or financial relationships that could be construed as a potential conflict of interest.
